# Sustained diabetes risk reduction after real life and primary health care setting implementation of the diabetes in Europe prevention using lifestyle, physical activity and nutritional intervention (DE-PLAN) project

**DOI:** 10.1186/s12889-017-4104-3

**Published:** 2017-02-15

**Authors:** Aleksandra Gilis-Januszewska, Jaana Lindström, Jaakko Tuomilehto, Beata Piwońska-Solska, Roman Topór-Mądry, Zbigniew Szybiński, Markku Peltonen, Peter E. H. Schwarz, Adam Windak, Alicja Hubalewska-Dydejczyk

**Affiliations:** 1 Chair and Department of Endocrinology, Jagiellonian University, Medical College, Kopernika 17, 31-501 Kraków, Poland; 2 Chronic Disease Prevention Unit, National Institute for Health and Welfare (THL), Helsinki, Finland; 3 Centre for Vascular Prevention, Danube-University Krems, Krems, Austria; 4 Department of Chronic Disease Prevention, National Institute for Health and Welfare, Helsinki, Finland; 5 Department of Public Health, University of Helsinki, Helsinki, Finland; 6 Diabetes Research Group, King Abdulaziz University, Jeddah, Saudi Arabia; 7 Dasman Diabetes Institute, Dasman, Kuwait; 8 Department of Epidemiology and Population Studies, Jagiellonian University Medical College, Krakow, Poland; 9 Department for Prevention & Care of Diabetes, Medical Clinic Unit III, University Clinic Carl Gustav Carus at Technical University DreSDen, DreSDen, Germany; 10 Department of Family Medicine, Chair of Medicine and Gerontology, Jagiellonian University Medical College, Krakow, Poland

**Keywords:** Diabetes type 2 prevention, Lifestyle intervention, Diet, Physical activity, Risk factors, Real life setting

## Abstract

**Background:**

Real life implementation studies performed in different settings and populations proved that lifestyle interventions in prevention of type 2 diabetes can be effective. However, little is known about long term results of these translational studies. Therefore, the purpose of this study was to examine the maintenance of diabetes type 2 risk factor reduction achieved 1 year after intervention and during 3 year follow-up in primary health care setting in Poland.

**Methods:**

Study participants (*n* = 262), middle aged, slightly obese, with increased type 2 diabetes risk ((age 55.5 (SD = 11.3), BMI 32 (SD = 4.8), Finnish Diabetes Risk Score FINDRISC 18.4 (SD = 2.9)) but no diabetes at baseline, were invited for 1 individual and 10 group lifestyle counselling sessions as well as received 6 motivational phone calls and 2 letters followed by organized physical activity sessions combined with counselling to increase physical activity.

Measurements were performed at baseline and then repeated 1 and 3 years after the initiation of the intervention.

**Results:**

One hundred five participants completed all 3 examinations (baseline age 56.6 (SD = 10.7)), BMI 31.1 (SD = 4.9)), FINDRISC 18.57 (SD = 3.09)). Males comprised 13% of the group, 10% of the patients presented impaired fasting glucose (IFG) and 14% impaired glucose tolerance (IGT). Mean weight of participants decreased by 2.27 kg (SD = 5.25) after 1 year (*p* = <0.001). After 3 years a weight gain by 1.13 kg (SD = 4.6) (*p* = 0.04) was observed. In comparison with baseline however, the mean total weight loss at the end of the study was maintained by 1.14 kg (SD = 5.8) (ns). Diabetes risk (FINDRISC) declined after one year by 2.8 (SD = 3.6) (*p* = 0.001) and the decrease by 2.26 (SD = 4.27) was maintained after 3 years (*p* = 0.001). Body mass reduction by >5% was achieved after 1 and 3 years by 27 and 19% of the participants, respectively.

Repeated measures analysis revealed significant changes observed from baseline to year 1 and year 3 in: weight (*p* = 0.048), BMI (*p* = 0.001), total cholesterol (*p* = 0.013), TG (*p* = 0.061), fasting glucose level (*p* = 0.037) and FINDRISC (*p* = 0.001) parameters. The conversion rate to diabetes was 2% after 1 year and 7% after 3 years.

**Conclusions:**

Type 2 diabetes prevention in real life primary health care setting through lifestyle intervention delivered by trained nurses leads to modest weight reduction, favorable cardiovascular risk factors changes and decrease of diabetes risk. These beneficial outcomes can be maintained at a 3-year follow-up.

**Trial registration:**

ISRCTN, ID ISRCTN96692060, registered 03.08.2016 retrospectively

## Background

Randomized control trials (RCT) performed in different populations have demonstrated up to 60% reduction in type 2 diabetes incidence through lifestyle intervention which leads to dietary and physical activity changes [1–4]. Furthermore, the effect of interventions in RCT setting has been shown to continue up to 20 years with 34–43% diabetes risk reduction [5–8]. Typically, the interventions in these clinical efficacy trials have been intensive and thus costly. Therefore, EU initiated and sponsored the DE-PLAN project (Diabetes in Europe: Prevention Using Lifestyle, Physical Activity and Nutritional Intervention) as a real life implementation study in 17 countries in Europe [9]. The aim of the project was to assess the reach of the programs, adoption and implementation in diverse real life settings, but also to create a network of trained and experienced professionals to continue diabetes prevention across Europe [9]. Indeed, real life implementation studies conducted in different settings and populations proved that less intensive, lower budget lifestyle interventions can be effective [10–21]. However, little is known about the long term results of these translational studies. Therefore, the purpose of this study was to examine the maintenance of risk factor reduction during 3 year follow-up in real life, primary health care setting in Poland.

## Methods

### Design

The intervention conducted in the DE-PLAN project was based on the principles of the Diabetes Prevention Study [1, 9]. Given that the efficacy of lifestyle modification treatments has been well established by earlier diabetes prevention trials, the need for an additional randomized controlled trial study design in the current program was regarded as unnecessary and unethical. A detailed description of the program performed in Poland, including the inclusion criteria, the characteristic of participants, methods, the intervention and one-year results has been published previously [10].

### Participants

The study was performed in 9 independent primary health care General Practitioners (GP) practices in Krakow. The study group consisted of everyday patients, city inhabitants, aged over 25. The inclusion criterion was high diabetes risk assessed with the Finnish Diabetes Risk Score (FINDRISC) > 14) (33% chance of developing diabetes within 10 years) [22], the exclusion criteria was either known diabetes or oral glucose tolerance test (OGTT) screening diabetes as well as known chronic disease which could affect the results of the study. Advertisements were placed alongside self-screening questionnaires in the GPs’ waiting rooms. In addition, patients with known risk factors were directly approached by nursing and medical staff. Out of 800 leaflets with the FRS questionnaire distributed in co-operating practices, 566 were completed. 368 respondents scored FRS >14; 275 agreed to undergo OGTT examination and subsequently 262 (with all measurements done) were invited to participate in the intervention. 175 participants completed the intervention and the final examination after 1 year and 113 completed follow-up examination after 3 years. 9 people (8 with complete measurements) who participated in the 3 year follow-up did not participate in 1 year examination. 105 patients took part in all 3 measurements (completers) while 79 did not participate in the 1 year and 3 year follow-up examinations (non-completers). The most commonly declared non-participation reason was shortage of time and inability to continue “time-consuming program”.

### Intervention

Lifestyle intervention implemented the principles of the Diabetes Prevention Study [1] and was based on reinforced behavior modification focusing on five lifestyle goals: loss of initial weight, reduced intake of total and saturated fats, increased consumption of fruit, vegetables and fiber and increased physical activity [1, 9, 10]. The intervention curriculum was created on the basis of written materials containing basic information about diabetes, diabetes prevention, diet, diet examples booklet and information about physical activity.

Well-trained nurses (2 nurses per one center), certified in diabetes prevention, delivered 10-month intervention. The initial intensive phase of intervention (4 months) consisted of 1 individual session followed by 10 group sessions (10–14 people), focusing on diet and physical activity changes. During each session printed educational materials related to the topic of the session were distributed. Social support was emphasized by the group setting and participants were also encouraged to invite their own social environment to lifestyle changes. A spouse or other family member could also participate in the sessions. After the initial 4 weeks of the intervention patients could take part in physical activity sessions twice a week (once a week – aqua aerobics; once a week – gymnastics or football). The following maintenance phase of the intervention (month: 4–10) following the intensive phase consisted of six motivational telephone sessions and two motivational letters received by the participants [1, 9, 10]. There were no other post-intervention contacts with the participants except measurements in year 1 and 3.

### Measurements

Patients were examined at baseline as well as after 12 and 36 months of the study. The examination procedure included: questionnaires (FINDRISC, baseline, clinical and lifestyle and quality of life) and biochemical tests including: fasting and 120’OGTT glucose, serum triglycerides, HDL and total cholesterol. Impaired Fasting Glucose (IFG) was defined as fasting plasma glucose concentration of 6.1 to 7.0 mmol/l. Impaired Glucose Tolerance (IGT) was defined as glucose plasma concentration of 7.80 to 11.0 mmol/l after oral administration of 75 g of glucose (OGTT), diabetes mellitus (DM) was defined as fasting glucose concentration of more than 7.0 mmol/l [23]. Body mass index (BMI) was calculated as weight (in light indoor clothes, kg) divided by height squared (m2), waist circumference was measured midway between the lowest rib and iliac crest, diastolic and systolic blood pressure were taken while sitting after 10 min rest.

### Ethics

This study followed the Good Clinical Practice guidelines and the guidelines of the Helsinki Declaration. The study protocol was approved by the Jagiellonian University Ethics Committee.

### Statistical analyses

The descriptive analyses are given in percentages (for categorical variables) and means with standard deviations (for continuous variables). The normality of distribution was assessed by skewness and kurtosis analysis. Differences between groups were assessed using chi-square and t-test for dependent groups (respectively for the type of data). For comparison of the 3 measurements the repeated measures ANOVA and main effect comparisons (pairwise t-tests) with Bonferroni correction was performed. All analyses were competed with SPSS v.20. *P*-value of < 0.05 was considered as the level of statistical significance.

## Results

105 middle aged participants (age 56.6 (SD = 10.7)), slightly obese (BMI 31.11 (SD = 4.9)), with high risk of developing diabetes (FRS 18.57 (SD = 3.09) completed all 3 examinations.

### Baseline characteristics of completers vs non-completers

Baseline characteristics of completers (*n* = 105) vs non-completers (*n* = 79) is given in Table 1. At baseline, non-completers in comparison with completers were heavier (89.7 vs 82.85 kg), had higher BMI (32.66 vs 31.11 kg/m2) and waist circumference 101.23 vs 96.67 cm) (p for all <0.05). Additionally non-completers had higher systolic and diastolic blood pressure (134.8 vs 130.7 (*p* = 0.051) and 83.48 vs 80.8 (*p* = 0.060), respectively) as well as higher fasting and OGTT glucose level (5.55 vs 5.22 (*p* = 0.01) and 7.1 vs 5.77 mmol/l (*p* < 0.001), respectively. Completers had normal glucose tolerance (NGT) more often than non-completers (76% vs 63% (*p* = 0.04)). 13% of the completers and 31.9% of the non-completers were men (*p* = 0.004). No other biochemical, anthropometric and sociodemographic differences between completers and non-completers were found.

**Table 1:** Baseline characteristic of completers (*n* = 105) and non-completers (*n* = 79) of 1 year and 3 year examination

	Completers		Non-completers		*P* value
	Mean/%	SD	Mean	SD	
Age	56,55	10,66	54,35	12,42	0,199
% men	13		32		0.004
Weight (kg)	82,85	15,20	89,70	15,73	0,003
BMI (kg/m2)	31,10	4,93	32,66	4,58	0,031
WC (cm)	96,67	11,41	101,23	9,41	0,004
SBP (mmHg)	130,72	14,09	134,80	13,77	0,051
DBP (mmHg)	80,80	8,97	83,47	10,08	0,060
Fasting glucose (mmol/l)	5,22	0,72	5,55	1,02	0,010
2-h OGTT glucose (mmol/l)	5,78	1,75	7,11	3,04	0,000
TCH (mmol/l)	5,55	0,94	5,81	1,30	0,127
HDL (mmol/l)	1,39	0,35	1,36	0,36	0,564
TG (mmol/l)	1,77	1,38	2,37	2,51	0,040
FINDRISC	18,57	3,09	18,82	2,95	0,584
NGT%	76		63		0.04
Education: basic % Middle % High %	46 42 12		40 44 15		0.740
Married/having a partner% Single/widow%	66 34		72 28		0.22
Working Retired Not working	41 53 6		53 41 6		0.219
Smoking currently	15		23		0.133
History of increased glucose					
History of hypertension	71		76		0.256
History of hyperlipidaemia	46		51		0.304
History of depression					
History of CVD					
Family history of diabetes	62		67		0.285

Key: *BMI* body mass index, *SBP* systolic blood pressure, *DBP* diastolic blood pressure, *OGTT* oral glucose tolerance test, *TCH* total cholesterol, *HDL* high density lipoprotein, *TG* triglycerides, *NGT* normal glucose tolerance, *history of CVD* history of cardiovascular disease

### Clinical outcomes for completers

Clinical and metabolic characteristics of completers from baseline to year 1 and 3 is given in Table 2. Using repeated measures statistical analysis, we found significant changes in the following parameters: weight (*p* = 0.048), BMI (*p* = 0.001), glucose level (*p* = 0.037), total cholesterol (*p=*0.013), TG (*p=* 0.061) and FINDRISC (*p* = 0.001) Mean weight decreased by 2.27 kg (SD = 5.24) after one year (*p* = 0.001). After 3 years a weight gain of 1.13 kg (SD = 4.6) (*p* = 0.0405) was noted. Nonetheless, the mean weight was still lower compared to baseline by 1.14 kg (SD = 5.8) (ns).

**Table 2:** Repeated measures analysis of clinical and metabolic characteristic from baseline to year 1 and 3 in the Diabetes in Europe Prevention Using Lifestyle, Physical Activity and Nutritional Intervention (DE-PLAN) project (*n* = 105)

		Baseline		Change from baseline to year 1		Change from baseline to year 3		*P* value			
	No.	Mean	SD	Mean	SD	Mean	SD	Baseline vs after 1 year	Baseline vs after 2 years	1 year vs 3 years	Repeated measures all changes
Weight (kg)	105	82,85	15,20	−2,27	5,25	−1,14	5,83	0,000	0,143	0,041	0,048
BMI (kg/m2)	105	31,10	4,93	−0,84	1,91	−0,35	2,18	0,000	0,000	0,018	0,000
WC (cm)	105	96,67	11,41	−3,95	5,71	−0,62	7,40	0,000	1,000	0,000	0,100
SBP (mmHg)	105	130,72	14,09	−1,97	15,58	0,11	16,50	0,593	1,000	0,472	0,313
DBP (mmHg)	105	80,80	8,97	−1,36	9,10	−0,56	10,25	0,385	1,000	0,955	0,579
Fasting glucose (mmol/l)	105	5,22	0,72	0,17	0,67	0,13	0,68	0,066	0,067	1,000	0,037
2-h OGTT glucose (mmol/l)	105	5,78	1,75	0,29	2,12	0,21	2,45	0,485	1,000	1,000	0,373
TCH ( mmol/l)	105	5,55	0,94	−0,26	1,16	−0,29	1,03	0,065	0,016	1,000	0,013
HDL ( mmol/l)	105	1,39	0,35	−0,05	0,28	−0,05	0,54	0,027	0,053	1,000	0,352
TG ( mmol/l)	105	1,77	1,38	−0,14	1,33	−0,23	1,23	0,130	0,120	0,560	0,061
FINDRISK	105	18,57	3,09	−2,81	3,64	−2,27	4,28	0,000	0,000	0,503	0,000

Key: *BMI* body mass index, *SBP* systolic blood pressure, *DBP* diastolic blood pressure, *OGTT* oral glucose tolerance test, *TCH* total cholesterol, *HDL* high density lipoprotein, *TG* triglycerides

The same trend of changes after 1 and 3 years was observed for BMI (*p* < 0.001 for both time points). Total cholesterol level diminished after one year by 0.26 mmol/l (SD = 1.16) (*p* = 0.065) and by 0.29 mmol/l (SD = 1.03) after 3 years (*p=* 0.016). TG decreased after one year by 0.14 mmol/l (SD = 1.33) (ns) and by 0.23 (SD = 1.22) (ns) after 3 years. FINDRISC went down after one year by 2.8 (SD = 3.6) (*p* = 0.001) and by 2.26 (SD = 4.27) after 3 years (*p* = 0.001). We also observed an increase of fasting glucose after one year by 0.17 mmol/l (SD = 0.67) (*p* = 0.066) and by 1.12 mmol/l (SD = 0.68) (*p* = 0.067) after 3 years.

At baseline 76% of participants had NGT, 10% IFG and 14% IGT. After 1 year of the study 73% of patients had NGT, 2% DM, 5% IFG and 20% IGT. After 3 years 74% of participants had NGT, 7% DM, 9% IFG and 11% IGT.

After 1 year 15% of baseline NGT patients converted to IFG or IGT and 1% to DM. After 3 years 19% of baseline NGT patients converted to IFG or IGT and 1% to DM. 10% and 20% of baseline IFG patients converted to DM after 1 and 3 years, respectively. Among baseline IGT participants none developed DM after 1 year but 27% converted to DM after 3 years of the study. 2 people with DM diagnosed after 1 year participated in the 3 year examination ( treated with diet only). They were categorized as DM again. After one year 27% participants lost weight by > 5%, 43% by < 5% and 31% did not change or increase body mass. After 3 years 19% participants maintained lower weight decreased by > 5%, 37% decreased by < 5%, and 44% did not change weight or increased body mass.

## Discussion

The results of this study show that type 2 diabetes prevention through lifestyle intervention in a primary health care setting is feasible and effective, and the results can be maintained during long-time observation. The evidence from RCTs confirmed that through lifestyle intervention including dietary modification, weight loss and physical activity, the reduction in type 2 diabetes incidence might be maintained long after the intervention. [5–8]. While there is evidence from RCTs, there are only a few long-time observations of implementation studies [21, 24]. In our real life implementation study conducted by trained nurses we demonstrated a modest weight reduction, by 2.27 kg, after 1 year of intervention. The change was subtle, however, it was accompanied by a reduction of total blood cholesterol, triglycerides as well as a lowered diabetes risk. Results achieved at one year were further maintained at 3 year follow-up.

Similar results were achieved in The Good Ageing in Lahti Region (GOAL) Lifestyle Implementation trial. This study was also designed for primary health care setting with lifestyle and risk reduction objectives based on the DPS. Similarly, the intervention was conducted by study nurses and consisted of 6 group counselling sessions [14, 21]. Patients were examined in year 1 of the intervention and after 3 years of the follow-up. The results after one year were also modest with the mean body mass reduction of 0.8 (SD = 4.5 kg) followed by modest but significant reduction in waist circumference, fasting glucose and total cholesterol. The 3 year follow-up body weight reduction was 1.0 (SD = 5.6 kg) which is comparable to our results. Furthermore, total cholesterol decrease and triglycerides reduction were also maintained after 3 years.

In our study the conversion rate to diabetes in all participants (baseline IGT, IFG and NGT) reached 2% after 1 year and 7% after 3 years. Data concerning the conversion rate are not easy to compare between different studies as various design, time of observation and risk of intervened people were applied. In the GOAL study, for example, the conversion rate from IGT to type 2 diabetes was 12% at 3 year follow-up while in the DPS study, where all participants had baseline IGT, the conversion rate was 9% in the intervention and 20% in the control group [4, 21]. The lower conversion rate in our study might be explained by lower risk level at baseline. In the DPS all participants had IGT at baseline, while in our study only 10% exhibited IFG and 14% IGT. However, the very low conversion rate to diabetes in our study should be regarded as an evidence of successful short and long term intervention.

In a review focusing on translational lifestyle interventions, Johnson et al. concluded that effectiveness could not be easily demonstrated with clinical parameters, such as blood glucose or T2DM risk [19].

Given the relatively short follow-up time and small sample size, real life prevention studies may have sufficient statistical power to measure change in weight rather than T2DM incidence [19]. Therefore, weight loss in these studies can be regarded as a marker for potential prevention in the long-term [19, 20].

Additionally, the reduction in FINDRISC score in our study, which changed from 18.57 at baseline to 15.76 at year 1 and 16.30 at year 3 can be used as a surrogate marker for diabetes risk reduction.

In the DPP study, weight loss was reported to be the dominant factor in T2DM prevention, with a loss of 5 kg explaining the 55% reduction incidence of T2DM over 3 years follow-up [24]. In the RCTs weight loss was correlated with the intensity of the delivered program. It is important to remember that intervention given in RTCs is typically incomparable to translational studies, which typically provide a less intensive, low budget intervention adapted to local and cultural possibilities. Consequently, as seen also in our study, the achieved weight reduction is usually modest compared with RCTs. Also, the percentage of people who lost > 5% of initial weight was substantially lower in our study than in the DPS or DPP studies (27% after one year and 19% after 3 years of the follow-up). However, modest weight reduction in our study should be regarded as a measure of success. In general population there is a weight increase by 0.5 kg per year, thus even small weight reduction or weight maintenance should be considered important achievements in diabetes prevention [25].

Although in our study there was a weight regain of 1.13 kg after 3 years, the weight decrease by 1.14 kg was still maintained even though the metabolic changes observed after intervention were still present at 3 year follow-up. According to the DPS, body weight during follow-up increased gradually in both groups but a statistically significant difference between the study groups prevailed [6]. Moreover, both metabolic improvements and lifestyle changes continued over time in those who were intervened [6].

In the meta-analysis of 22 real life implementation studies conducted by Dunkley et al. the mean proportion of weight lost (%) at 12 months follow-up was 2.6%. It was concluded that despite the drop-off in intervention effectiveness in translational studies, the modest level of weight loss found in the analysis is still likely to have a clinically meaningful effect on diabetes incidence. The rate of progression to diabetes was calculated 34 per 1000 person-years which suggests that the real world lifestyle intervention studies achieved lower diabetes progression rates in comparison to natural progression rates in high risk individuals [20].

Some limitations of our study need to be discussed. The participants in our study were volunteers, and similarly to many other studies, our study predominantly attracted women, who accounted for 87% of participating in both examinations. Thus, our results might not be generalized in reference to both sexes. In addition, rather modest results obtained in our study might be influenced by female sex domination, whose success in previous diabetes prevention studies was meager when compared to men [26]. It also implies the need for further studies on sex specific mechanism in real life lifestyle interventions. Our study also confirmed that people who participate in epidemiological studies have a healthier profile, are less obese and have better blood pressure and biochemical profiles than non-attenders, therefore the results of the intervention might be less obvious in those baseline healthier people [27]. In our study there were no particular socioeconomic differences between completers and non-completers. However low socioeconomic status could be related to less frequent use of healthcare services despite poorer health status [28]. These observations highlight the need to develop lifestyle interventions further in order to increase participation of males and particularly those who are at high risk [18, 20].

A modified program based on the DE-PLAN sponsored by the local self-government is being continued in Krakow. Methodology, results and experience from the DE-PLAN project were used in the preparation of the European guidelines for the prevention of type 2 diabetes and the toolkit for diabetes prevention in Europe [29, 30]. There are also some other European initiatives on diabetes type 2 prevention and early prevention of diabetes complications following the DE-PLAN project: the IMAGE (Development and Implementation of a European Guideline and Training Standards for Diabetes Prevention), the MANAGE CARE (Active Ageing with Type 2 Diabetes as Model for the Development and Implementation of Innovative Chronic Care Management in Europe) and the ePREDICE (Early Prevention of Diabetes Complications in People with Hyperglycaemia in Europe) projects (www.idf.org).

## Conclusions

Type 2 diabetes prevention in primary health care setting through lifestyle intervention delivered by trained nurses leads to modest weight reduction, which is accompanied by favorable cardiovascular risk factors changes and diabetes risk reduction. These beneficial outcomes can be maintained at a 3-year follow-up.

## Key messages

Type 2 diabetes prevention in high risk individuals subject to lifestyle intervention may provide long-term benefits in biological parameters such as body weight and blood lipids.

## Abbreviations

CVD: Cardiovascular disease
DA: QUING the China da qing diabetes prevention study
DE-PLAN: Diabetes in Europe: prevention using lifestyle, physical activity and nutritional
DM 2: Diabetes mellitus type 2
DPP: Diabetes prevention program
DPS: Diabetes prevention study
IFG: impaired fasting glucose
IGT: Impaired glucose tolerance intervention
NGT: Normal glucose tolerance
OGTT: Oral glucose tolerance test
RCT: Randomized control studies
